# Supplementary material for: Conformational dynamics in TRPV1 channels reported by an encoded coumarin amino acid
Source: eLife. 2017 Dec 5;6:e28626. doi: 10.7554/eLife.28626 (PMC5716661; doi:10.7554/eLife.28626)
Supplement: Transparent reporting form [file elife-28626-transrepform.pdf]

eLIFE
1st Floor
24 Hills Road
Cambridge CB2 1JP, UK
P 01223 855340
W elifesciences.org
T @elife

# *eLife's* transparent reporting form

We encourage authors to provide detailed information *within their submission* to facilitate the interpretation and replication of experiments. If you have any questions, please contact us: editorial@elifesciences.org.

**Sample-size estimation**

- You should state whether an appropriate sample size was computed when the study was being designed
- You should state the statistical method of sample size computation and any required assumptions
- If no explicit power analysis was used, you should describe how you decided what sample (replicate) size (number) to use

Please outline where this information can be found within the submission (e.g., page numbers or figure legends), or explain why this information doesn't apply to your submission:

No estimation or computation was made to determine the sample size. Because of the lack of such estimation, and the technical complexity of the experimental procedure, all the data obtained was considered for the analysis.

**Replicates**

- You should report how often each experiment was performed
- You should include a definition of biological versus technical replication
- The data obtained should be provided and sufficient information should be provided to indicate the number of independent biological and/or technical replicates
- If you encountered any outliers, you should describe how these were handled
- Criteria for exclusion/inclusion of data should be clearly stated
- High-throughput sequence data should be uploaded before submission, with a private link for reviewers provided (these are available from both GEO and ArrayExpress)

Please outline where this information can be found within the submission (e.g., page numbers or figure legends), or explain why this information doesn't apply to your submission:

eLife Sciences Publications, Ltd is a limited liability non-profit non-stock corporation incorporated in the State of Delaware, USA, with company number 5030732, and is registered in the UK with company number FC030576 and

A large set of experiments was performed using different techniques. Most experiments are based on the observation and analysis of fluorescence fluctuations of single emitters. When the levels of expression allow, several puncta can be discriminated as potential single emitter. From this subset, just about 30 % bleached on one step during the illumination period. Given the low efficiency of amber codon suppression, obtaining healthy expressing cells was the critical step. From the few cells expressing not all of them were attached to the glass and this is a requirement for TIRF experiments. The imaging experiments performed are really hard to put together and for that reason data collection was tedious. The modest number of experiments makes us to do two things, show raw data on each figure and present the full data set when possible. As an example, supplemental figure 2 contains the full data set used for the unitary transition analysis (critical on the present work), without considering the existence of potential outliers (the sample was too small to do that). We fit the data all together in that summary figure (figure 3, SF2) and we observed that one order of magnitude difference in concentration was needed to make the average significantly different ($p<0.01$).

**Statistical reporting**

- Statistical analysis methods should be described and justified
- Raw data should be presented in figures whenever informative to do so (typically when N per group is less than 10)
- For each experiment, you should identify the statistical tests used, exact values of N, definitions of center, methods of multiple test correction, and dispersion and precision measures (e.g., mean, median, SD, SEM, confidence intervals; and, for the major substantive results, a measure of effect size (e.g., Pearson's r, Cohen's d)
- Report exact p-values wherever possible alongside the summary statistics and 95% confidence intervals. These should be reported for all key questions and not only when the p-value is less than 0.05.

eLIFE
1st Floor
24 Hills Road
Cambridge CB2 1JP, UK
P 01223 855340
W elifesciences.org
T @elife

Please outline where this information can be found within the submission (e.g., page numbers or figure legends), or explain why this information doesn't apply to your submission:

Distribution histograms were computed to discriminate between single bleaching transitions when counting emitters (figure 2d). When fitting to exponential decays (figure 3a and d), hill function (Figure 3e), and Boltzmann distributions (figures 1d and 3f) we considered $\chi^2$ as a measurement of the goodness of the fit. A *p*-value of 0.05 was considered significant when comparing the modes and p<0.01 when analyzing the mean values used on the dose-response curve (figure s2). When averaged, data is shown with the correspondent SEM. All figures show raw data for the readers to evaluate. The number of experiments performed for the different sets were as follows.
Whole cell electrophysiology. The GV curves were not analyzed further because of the small differences observed. We are not aiming to describe such details here. Each curve corresponds to the average of 6 individual experiments.
Single photon counting. Individual cells on one day of transfection contribute with several puncta, considered replicates. Therefore the histogram presented on figure 1g corresponds to n=1 but comes from the average of several individual histograms (in this case 5 on each curve). The final statistical procedure was done by comparing the mode of the different populations, n=4 for each condition.
Photobleaching. Each individual puncta measured went through this procedure. We considered the histograms to discriminate the different populations of molecules. Figure 2b depicts the different set of data we handled.
Ensemble average. We repeated this measure three times. It includes all the bright spots on one cell in each case. One individual example (raw data) is shown on figure 2c. No statistical procedure was performed in this case.
Noise analysis. Seven different puncta on each condition was analyzed separately and each exponential decay function extracted. The average of the different exponential decays obtained is presented (figure 3a). N=7 for each condition.
Idealization and unitary transitions. The idealization was performed first by checking that the base level was similar to the background noise, then setting a defined threshold on 2 standard deviations of background noise average (figure 3b). The comparison between the dwell times was performed in paired experiments (0 versus 3 uM, n=5). A raw dataset is presented on figure 3c. The full data set for the DR curve and its fitting is available on figure 3 (SF2) and the mean value (SEM) was depicted (in different forms) on figures 3e and f.

No statistical method was performed for the theoretical work shown on figure 4.

eLife Sciences Publications, Ltd is a limited liability non-profit non-stock corporation incorporated in the State of Delaware, USA, with company number 5030732, and is registered in the UK with company number FC030576 and

(For large datasets, or papers with a very large number of statistical tests, you may upload a single table file with tests, Ns, etc., with reference to page numbers in the manuscript.)

**Additional data files ("source data")**

- We encourage you to upload relevant additional data files, such as numerical data that are represented as a graph in a figure, or as a summary table
- Where provided, these should be in the most useful format, and they can be uploaded as "Source data" files linked to a main figure or table
- Include model definition files including the full list of parameters used
- Include code used for data analysis (e.g., R, MatLab)
- Avoid stating that data files are "available upon request"

Please indicate the figures or tables for which source data files have been provided:

| |
|---|
| A file containing all the experimental data used to prepare the figure set is available here: https://www.dropbox.com/s/xlyisdrhj4tm3kd/Steinberg%20et%20al.opj?dl=0 |
